# Platelet-rich plasma for jumper's knee: a comprehensive review of efficacy, protocols, and future directions

**DOI:** 10.1007/s00590-023-03713-9

**Published:** 2023-09-05

**Authors:** Francesco Bosco, Riccardo Giai Via, Fortunato Giustra, Alessandro Ghirri, Giorgio Cacciola, Alessandro Massè

**Affiliations:** 1 Department of Orthopaedics and Traumatology, University of Turin, CTO, Via Zuretti 29, 10126 Turin, Italy; 2 Department of Orthopaedics and Traumatology, Ospedale San Giovanni Bosco di Torino - ASL Città di Torino, Turin, Italy

**Keywords:** Platelet-rich plasma, Jumper's knee, Patellar tendinopathy, Efficacy, PRP, Knee

## Abstract

**Purpose:**

This comprehensive review evaluates the current state of platelet-rich plasma (PRP) treatment for jumper's knee, also known as patellar tendinopathy. The aim is to assess the efficacy of PRP as a therapeutic option compared to other available procedures, investigate the benefits and potential drawbacks of PRP infiltration, and provide insights into the optimal protocols for PRP preparation and administration.

**Methods:**

A comprehensive literature search of English articles published up to June 2023 was conducted using PubMed and Scopus databases. Studies evaluating PRP for treating jumper's knee or patellar tendinopathy were analyzed to assess the current state of research in this field.

**Results:**

PRP has demonstrated promising results in promoting cellular remodeling and accelerating the healing process in the jumper's knee. It shows potential benefits in pain reduction, improved function, and accelerated recovery. However, the efficacy of PRP varies depending on patient characteristics, disease severity, and the specific administration methodology. Establishing standardized PRP preparation and administration protocols are necessary to optimize its effectiveness. Further research is needed to define appropriate patient selection criteria and refine the application of PRP therapy in patellar tendinopathy management.

**Conclusion:**

Jumper's knee is commonly managed conservatively, but there is a lack of consensus on further treatment options. PRP treatment holds promise in promoting tissue healing and repair. However, standardized protocols for PRP preparation and administration, as well as optimal dosage and number of injections, require further investigation to enhance its efficacy. Continued research efforts are necessary to ascertain the precise role of PRP and its refinement in the management of patellar tendinopathy.

## Introduction

Jumper's knee, also referred to as patellar tendinopathy, is a condition that affects the extensor apparatus at the patellar level, commonly seen in professional athletes engaged in activities that involve jumping, sprinting, and sudden changes of direction on hard surfaces while bearing significant loads [1, 2]. Lian et al. reported an incidence rate of approximately 14% in athletes, with recurrence rates as high as 45% in volleyball players and 32% in basketball players [3, 4]. The condition is more prevalent in males and presents as insidious onset pain at the proximal insertion site of the quadriceps tendon over the patella (20% of cases), pain at the inferior pole of the patella (70% of cases), or less commonly, pain at the tibial tuberosity (10%), which may increase during physical activity and digital pressure [4–6]. The diagnosis of the jumper's knee is clinical. However, diagnostic tools such as ultrasonography (US) or magnetic resonance imaging (MRI) may confirm the diagnosis by revealing a smaller cross-sectional area of the tendon and abnormalities of the posterior border of the patellar tendon and Hoffa's body caused by an inflammatory pattern [7].

Conservative treatment is the first-line approach for jumper's knee, as for most tendinopathies. This therapy includes functional rest, ice, nonsteroidal anti-inflammatory drugs (NSAIDs), and sports footwear with soft insoles [8, 9]. Physical therapy to strengthen the flexibility of the thigh flexors and the quadriceps, particularly the vastus medialis, plays a crucial role in recovery. In literature, eccentric, concentric, and isometric training protocols have shown positive results [10, 11]. However, if physiotherapy fails, there is no consensus in the literature on further therapy. Modern therapeutic options include intratendinous injections of substances believed to inhibit neovascularization, such as corticosteroids (which can cause collagen sclerosis and long-term damage to the tendon), or of substances that relieve pain by other pathways (acupuncture, extracorporeal shock wave therapy [ESWT]) or that stimulate tendon repair (platelet-rich plasma [PRP]) [12–15]. PRP is an autologous blood fraction rich in platelets and their associated growth factors (GFs). In numerous in vitro and in vivo studies, PRP has demonstrated increased cellular remodeling effects and reduced healing time due to the release of these growth factors [16–18].

This study aims to define state of the art in patients with jumper's knee undergoing PRP infiltration to understand whether it is a viable treatment option compared with other available procedures to understand the benefits and potential disadvantages of PRP infiltration for jumper's knee.

## Search strategy

We conducted a literature review using the PubMed and Scopus databases to identify studies evaluating PRP use for treating jumper's knee or patellar tendinopathy. The search was limited to articles published in English up to June 2023.

## PRP in jumper’s knee—state of the art

**Fig. 1:**
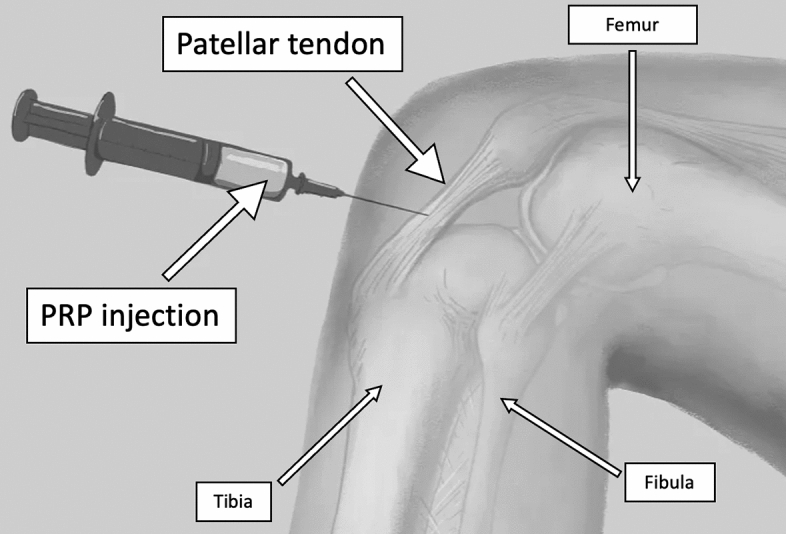
PRP injection is administered via infiltration into the patellar tendon to treat the jumper's knee. *PRP* platelet-rich plasma

Jumper's knee is a complex condition that presents a challenge in terms of effective treatment [8, 9]. Initial management typically involves conservative approaches, such as functional rest, application of ice, the use of nonsteroidal anti-inflammatory drugs (NSAIDs), and the adoption of sports footwear with soft insoles [8, 9]. Additionally, physical therapy focused on enhancing the flexibility of the thigh flexors and quadriceps muscles, with particular emphasis on the vastus medialis, plays a critical role in the recovery process [10, 11]. However, in cases where physiotherapy proves ineffective, there is currently no consensus in the literature regarding the appropriate next steps in treatment. One proposed procedure, in the literature [16–18], as a subsequent step is the administration of PRP injections (Fig. 1).

## Enhancing understanding of PRP biology and biomechanics in patellar tendinopathy

**Fig. 2:**
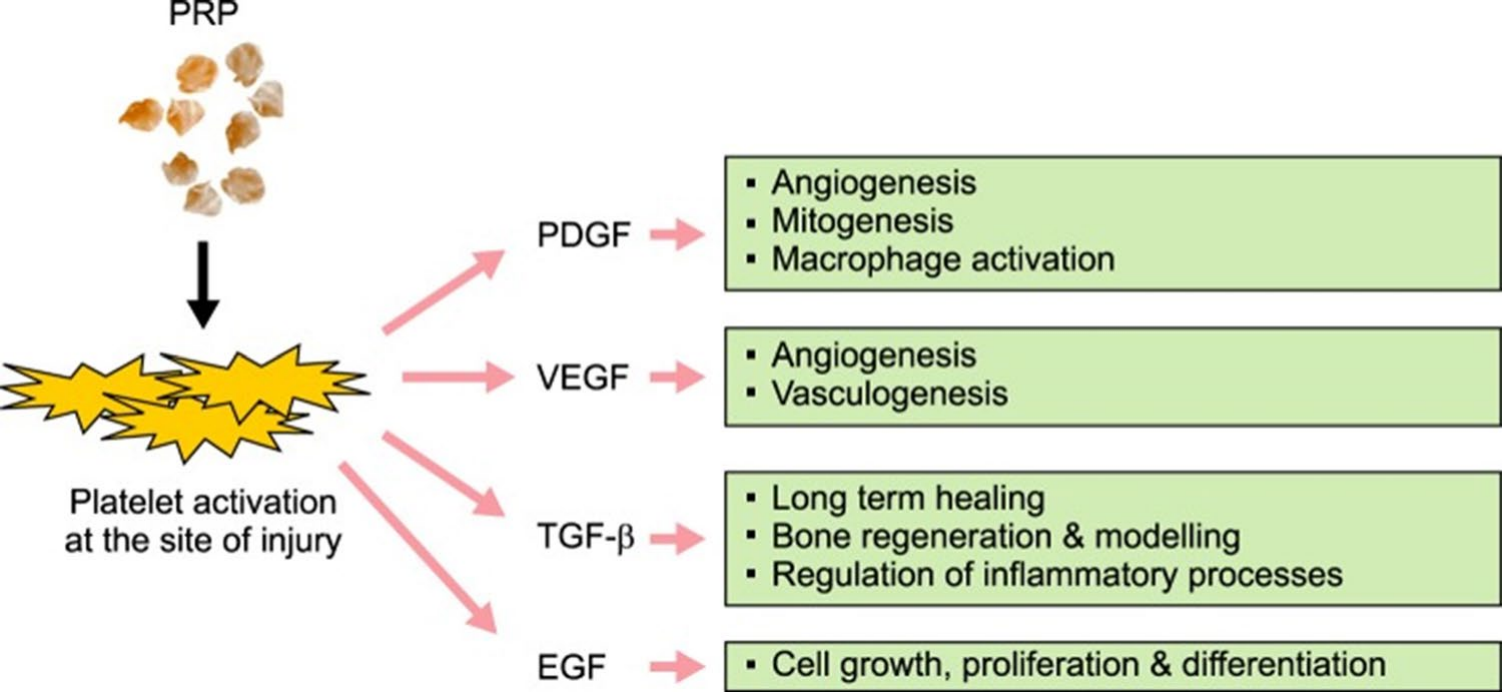
Mechanism of action of PRP. Wound healing and tissue regeneration can be accelerated at the site of tissue injury by various growth factors produced from activated platelets. *PRP* platelet-rich plasma; *PDGF* platelet-derived growth factor; *VEGF* vascular endothelial growth factor; *TGF-β* transforming growth factor β; *EGF* epidermal growth factor. The source is published under a Creative Commons License from Jain et al. [6]

Platelet-rich plasma (PRP) has recently gained considerable attention as a therapeutic option for patellar tendinopathy, offering a novel approach to accelerate healing and improve patient outcomes [16]. PRP is an autologous blood fraction that contains a high concentration of platelets and their associated growth factors (GFs), including platelet-derived GF (PDGF), vascular endothelial GF (VEGF), transforming GF-*β* (TGF-*β*), and epidermal GF (EGF) [6] (Fig. 2). These growth factors play crucial roles in regulating various cellular processes involved in tissue repair and regeneration [16–18]. When PRP is administered to the site of injury, the release of these GFs stimulates cellular remodeling and enhances the healing cascade. This promotes the recruitment of reparative cells, angiogenesis, and collagen synthesis, which contribute to tissue regeneration and improved structural integrity [17, 18]. The localized application of PRP has shown promising results in reducing pain, improving function, and accelerating the recovery process in patients with patellar tendinopathy [16–18]. One of the key advantages of PRP is its autologous nature, meaning it is derived from the patient's own blood, minimizing the risk of adverse reactions or immune responses. Additionally, PRP can be easily prepared in an outpatient setting, making it a convenient and cost-effective treatment option [18].

## PRP for jumper's knee: exploring healing acceleration and outcome improvement

In this literature review, several studies have explored the effectiveness of PRP as a treatment option for patellar tendinopathy, with mixed results [19–33]. Many studies have reported positive outcomes, such as improvements in knee pain and function, such as the study by Dragoo et al. [22] where they compared patients who underwent PRP with patients who underwent dry needling showing better results in the patients infiltrated for the VISA-P score at about 12 weeks, but with results superimposed on the control at 26 weeks. Another study showing promising results is the one by Vetrano et al. [14], where patients undergoing PRP infiltration demonstrated better results in the short and medium term than patients undergoing extracorporeal shock waves (ESWT). Several other studies in the literature [17, 27, 28, 30, 31] have shown good results after PRP infiltration with improvement in subjective scores, such as visual analogue scale (VAS), Tegner Activity Scale, Lysholm score, and International Knee Documentation Committee (IKDC) score. Good results have also been reported in studies where patients underwent physical therapy in addition to PRP infiltration [30, 31, 33]. However, other studies have shown little or no effect of PRP in treating patellar tendinopathy [22, 25, 26]. Krogh et al. [25] and Scott et al. [26] did not show superiority over saline injection in their studies of infiltrative treatment with one and, for Scott's study, even two doses of PRP. Other studies have also suggested a limited effect of PRP in treating patellar tendinopathy [14, 25]. In addition, interestingly, the study conducted by Filardo et al. [29] showed that patients with bilateral involvement and a long history of pain and limited function had significantly poorer results. PRP may be an effective therapeutic option for patellar tendinopathy, but the results are mixed and depend on patient characteristics, disease severity, and the PRP administration methodology. Further studies are needed to determine PRP’s outcomes more accurately in treating patellar tendinopathy.

## Improving protocols for PRP preparation and administration in patellar tendinopathy

**Fig. 3:**
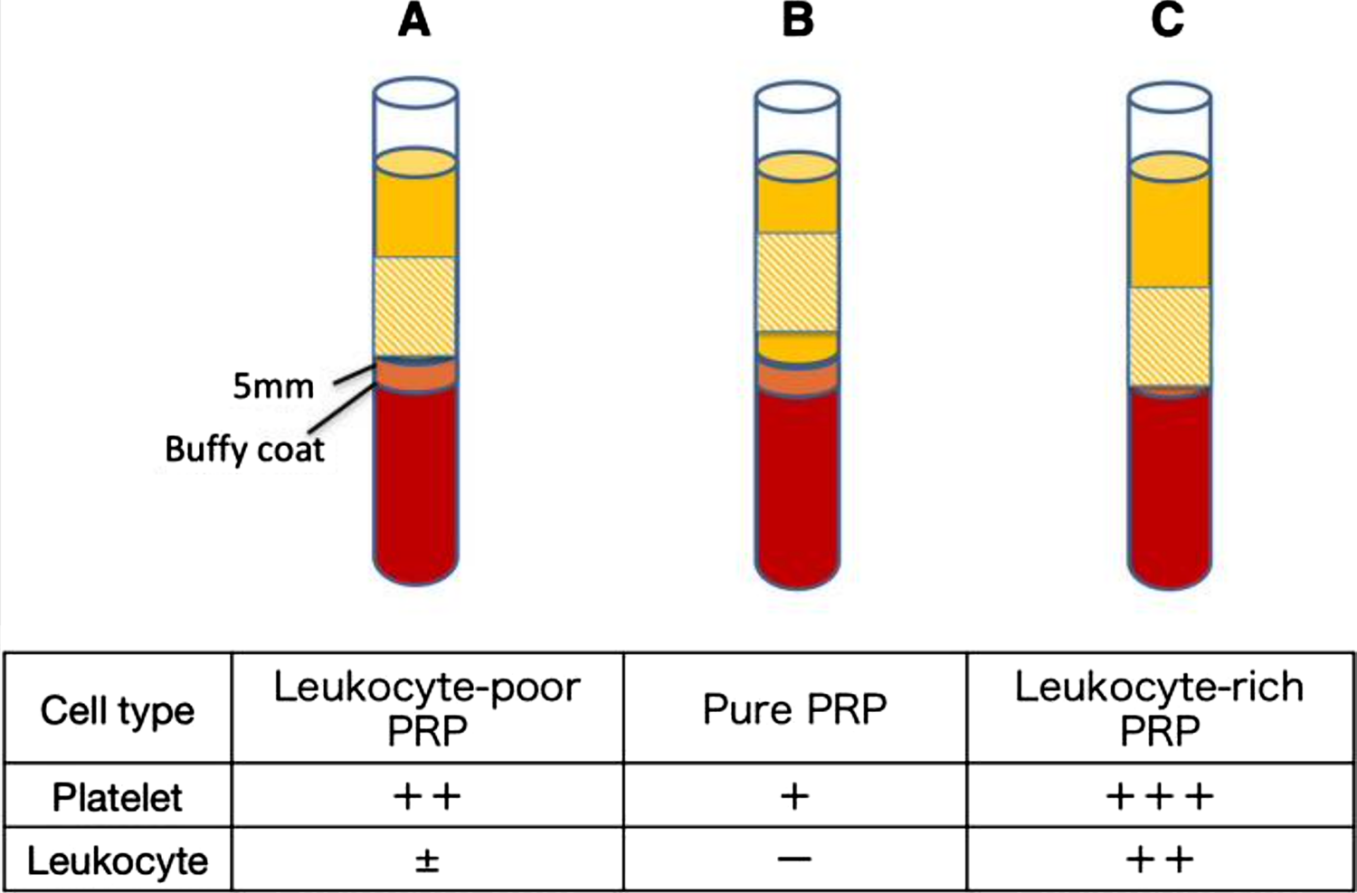
Scheme of the centrifuged blood with variants of PRP preparation. A line was drawn 5 mm above the buffy-coat layer in the separated blood, and the layer above this line was divided into two. The upper portion was designated as PPP, and the lower portion was designated as PRP **A** The target layer (shaded area) of the PRP system, containing LP-PRP. **B** When aspirating from above the target layer (shaded area), the platelet concentration obtained was relatively low, without contaminating leukocytes, representing purified PRP. **C** Aspirating from layers below the target layer (shaded area), platelet concentration increased, though leukocytes were retained, which was LR-PRP. *PPP* platelet-poor plasma; *PRP* platelet-rich plasma; *LP-PRP* leukocyte-poor-PRP; *LR-PRP* leukocyte-rich PRP The source is published under a Creative Commons License from Kikuchi et al. [24]

A major challenge in studying the efficacy of PRP in patellar tendinopathy is the lack of standardized protocols for preparing and administering PRP and the absence of protocol regarding uniformity on the correct amount and number of dosages. Numerous variations in the methods used to prepare and administer PRP may contribute to the mixed results observed in the literature [22–28]. Among the variants of PRP preparation described in the literature [22–28], we have those rich in leukocytes (LR-PRP) and those poor in leukocytes (LP-PRP) [24] (Fig. 3). Scott et al. [26], in a randomized controlled trial (RCT), analyzed the effects of LR-PRP and LP-PRP infiltration by comparing them with saline. They evidenced no superiority of one infiltrative method over the others, reporting only increased pain in the immediate post-infiltration period, most likely, according to the authors, induced by the leukocyte-mediated response in the group of patients receiving LR-PRP.

Among the variations in the methods of administration: In most of the studies analyzed, only one infiltration was performed [17, 22, 25, 26, 30, 31], two RCT studies and one prospective study, on the other hand, compared patients who received a single dose of PRP with those who received two with some contrasting results. Vetrano et al. [14] administered a second dose about seven days after the first one. They obtained good short- and medium-term results compared to the control arm undergoing extracorporeal shock waves (ESWT) [14]. In their study, they believe that these good results are due to the bleeding induced by the multiple punctures and by the growth factors (GFs) present in the PRP [14]. Also, Zayni et al. [32] highlighted that patients who received two injections had better results than those who received only one. At the same time, Kaux et al. [23] did not show superior results in patients undergoing two infiltrations. They, therefore, suggested administering a second dose of PRP where necessary at about three months in the case of partial recovery as a boost dose [23].

## Future direction

Further research is needed to establish standardized protocols for PRP administration to optimize its effectiveness. Another important consideration in the use of PRP for patellar tendinopathy is patient selection. It is crucial to identify patients who are most likely to benefit from PRP treatment. Factors such as disease severity, age, and comorbidities may influence the effectiveness of PRP in these patients. PRP is a promising therapeutic option for patellar tendinopathy, but its efficacy is mixed and dependent on patient characteristics, disease severity, and the PRP administration methodology. Further research is needed to establish standardized protocols for PRP administration and identify patients most likely to benefit from this treatment. With continued research, PRP may be a valuable addition to the therapeutic armamentarium for patellar tendinopathy.

## Strengths and limitations

The present study provides a comprehensive overview of jumper's knee and the potential benefits of PRP as a treatment option. It highlights the autologous nature of PRP, its ability to accelerate healing and improve outcomes through the release of GFs. The review also discusses the mixed results observed in studies evaluating PRP for jumper's knee, including positive outcomes such as pain reduction and improved function, as well as limitations and the need for standardized protocols. Overall, the study sheds light on the potential of PRP and the need for further research in optimizing its effectiveness for patellar tendinopathy.

Although this review provides a comprehensive analysis of the existing literature on the topic, there are several limitations that must be acknowledged. First, since this is a narrative review rather than a systematic review, the selection of studies evaluated in this review may have introduced biases due to the limited availability of research articles on certain aspects of the topic. Despite our efforts to include all relevant studies, it is possible that some important studies were overlooked or excluded due to our search criteria or other limitations. Second, the quality of the included studies varies, with some studies of higher quality than others. This may influence the overall conclusions drawn from this review and caution should be exercised in interpreting the results. Third, the scope of this review is limited to studies published up to the present date. As the field continues to evolve, new studies may emerge that challenge or extend the findings presented here. Finally, it is important to note that this review is a synthesis of the existing literature and, as such, does not involve the collection or analysis of original data. Therefore, the conclusions drawn from this review are limited by the quality and quantity of the studies included in the analysis. Although the use of PRP is promising, there are still several limitations to its use, such as the lack of standardization in PRP preparation, and further studies are needed to determine the effectiveness of PRP more precisely in treating patellar tendinopathy.

## Conclusions

Jumper's knee is a common condition affecting athletes engaged in jumping, sprinting, and sudden directional changes on hard surfaces. Conservative treatment is the first-line approach, but if physiotherapy fails, there is no consensus in the literature on further therapy. Platelet-rich plasma (PRP) has emerged as a promising therapy for jumper's knee, as it contains numerous growth factors and cytokines that promote tissue healing and repair, but further studies are needed to create standardized protocols for the preparation and administration of PRP and to understand the proper amount to infiltrate and to understand the most suitable number of injections.

## Data Availability

The dataset analyzed in this study is available from the corresponding author on reasonable request.
